# Isoliquiritigenin Alleviates Semen Strychni-Induced Neurotoxicity by Restoring the Metabolic Pathway of Neurotransmitters in Rats

**DOI:** 10.3389/fphar.2021.762290

**Published:** 2021-11-15

**Authors:** Lu Wang, Min Zhang, Jing Wen, Yalan Xiang, Xiaoyu Duan, Changwei Yu, Miao Yan, Bikui Zhang, Pingfei Fang

**Affiliations:** ^1^ Department of Pharmacy, Second Xiangya Hospital, Central South University, Changsha, China; ^2^ Institute of Clinical Pharmacy, Second Xiangya Hospital, Central South University, Changsha, China; ^3^ Third Hospital of Changsha, Changsha, China

**Keywords:** neurotransmitters, Semen Strychni, isoliquiritigenin, neurotoxicity, metabolic pathways, NMDA receptors

## Abstract

Acute neurotoxicity of Semen Strychni can result in sudden death in epilepsy. The detoxification method and mechanism of Semen Strychni acute poisoning have not been clarified. This experiment focused on the mechanism of Semen Strychni neurotoxicity and the alleviation effects of isoliquiritigenin. The rats were intraperitoneally injected with Semen Strychni extract (125 mg/kg), followed by oral administration of isoliquiritigenin (50 mg/kg) for 7 days. FJ-B staining was used to evaluate the degree of injury on hippocampus neurons. The concentration of monoamines, amino acids, and choline neurotransmitters, the Dopamine (DA) and 5-hydroxytryptamine (5-HT) metabolic pathway in the hippocampus, cerebellum, striatum, prefrontal cortex, hypothalamus, serum, and plasma were detected by LC-MS/MS. The expression of neurotransmitter metabolic enzymes [catechol-O-methyl transferase (COMT) and monoamine oxidase (MAO)] and neurotransmitter receptors [glutamate N-methyl-D-aspartic acid receptors (NMDARs) and gamma-aminobutyric acid type A receptor (GABRs)] were, respectively determined using ELISA and qRT-PCR. The results indicated that Semen Strychni induced neuronal degeneration in the hippocampal CA1 region. Meanwhile, Semen Strychni inhibited the mRNA expression of NMDAR1, NMDAR2A, NMDAR2B, GABRa1, GABRb2 and reduced the level of MAO, which disrupted the DA and 5-HT metabolic pathway. However, isoliquiritigenin reversed these effects. In summary, isoliquiritigenin showed alleviation effects on Semen Strychni-induced neurotoxicity, which could be attributed to restoring neurotransmitters metabolic pathway, most likely through the activation of NMDA receptors.

## Introduction

Semen Strychni, the dried seeds of *Strychnos nux-vomica L. (Loganiaceae)* with severe toxicity (Lu et al., 2020), has a long history of clinical applications in improving blood circulation, treating cancer and relieving rheumatic pain. It plays an essential role in the treatment of rheumatoid arthritis (Li et al., 2015). Studies indicated that strychnine and brucine, which account for about 70% of Strychnos alkaloids (SAs), are the main biologically active components of Semen Strychni (Lin et al., 2016). Semen Strychni is characterized by a narrow therapeutic window and individual differences, it has a prominent toxic effect, especially its neurotoxicity, as it can cross the blood-brain barrier (BBB). The blood-brain AUC ratios of brucine and strychnine were dose-related, limiting its clinical applications (Wu et al., 2012; Ren et al., 2018). Semen Strychni-induced neurotoxicity makes the central nervous system reflex center more sensitive to afferent stimuli and blocks postsynaptic inhibitory Glycine (Gly) receptors in the spinal cord and brain stem (Guo et al., 2018; Li et al., 2018).

Processing and compatibility are the most common methods to reduce the toxicity of Semen Strychni (Guo et al., 2018). Licorice (Glycyrrhizae Radix et Rhizoma) is the root of *Glycyrrhiza uralensis Fisch., Glycyrrhiza glabra L.* or *Glycyrrhiza inflata Bat.*, it is frequently used in traditional Chinese medicine. Licorice can reduce the toxicity of Semen Strychni without characterization of their active constituents (Gu et al., 2014; Zhang et al., 2018a; Ramalingam et al., 2018). Isoliquiritigenin (ISL), a flavonoid found in *Glycyrrhiza* glabrate, exerts neuroprotective effects, mainly through antioxidant, anti-inflammatory, anti-epilepsy, increasing energy metabolism (Zhan and Yang, 2006; Zhu et al., 2019a; Gao et al., 2020; Shi et al., 2020; Wang et al., 2020). However, little is known about the mechanism of action between ISL and Semen Strychni.

Neurotransmitters can be regarded as biomarkers of SAs-induced neurotoxicity (Sun et al., 2018; Vogt, 2019). It mainly including monoamines, amino acids, and choline. Glutamate (Glu) and γ-aminobutyric acid (GABA) are the primary excitatory and inhibitory amino acid neurotransmitters in the brain. An imbalance between Glu and GABA neurotransmission can cause epileptic seizures and irreversible neuronal damage (Yang et al., 2020; Akyuz et al., 2021). Gly is an inhibitory amino acid neurotransmitter that inhibits neuromotor (Fernando et al., 2015; Spiering, 2018). Due to this, an overdose of Semen Strychni developed widespread muscle spasms and convulsions (Naik and Chakrapani, 2009), severe cases due to clonic seizures resulting in tonic epilepsy and died of respiratory arrest (Malone et al., 1992). Acetylcholine (ACh) can act as a neurotransmitter or neuromodulator (Vogt, 2018), and it is involved in controlling breathing (Mutolo et al., 2011). As the study indicated, the level of ACh in rat serum of the Semen Strychni group showed a significant elevation, and Acetylcholinesterase (AchE) activity decreased (Li et al., 2018; Sun et al., 2018).

In neurotransmitter metabolism, Dopamine (DA) and Tryptophan (Trp) pathways are two critical metabolic pathways. In the DA pathway, the synthesis of DA, epinephrine (E), and norepinephrine (NE) are from Tyrosine (Tyr) by the enzyme Tyrosine Hydroxylase (TH), and further metabolized to 3,4-dihydroxyphenylacetic acid (DOPAC) and Homovanillic acid (HVA) via Catechol-O-methyl transferase (COMT) and Monoamine oxidase (MAO). They play an essential role in mood, behavior, and motor control (Matsubara et al., 2011; Dimic et al., 2017; Yao et al., 2020). In the Trp pathway, serotonin (5-hydroxytryptamine, 5-HT) is synthesized from Trp and biotransformed to 5-hydroxyindole acetic acid (5-HIAA) by MAO. Neurotoxicity can alter neurodevelopment and neurotransmitter metabolism-related genes expression patterns (Tian et al., 2021). Metabolic disorders in the 5-HT and DA pathways are associated with central nervous system diseases, such as sudden death in epilepsy (Wu et al., 2012; Comai et al., 2020). The above results are similar to the symptoms of Semen Strychni poisoning. Maybe Semen Strychni neurotoxicity is associated with these neurotransmitters.

In the present study, we evaluated the degree of neuronal damage in Semen Strychni extract (SSE) and the alleviating effect of ISL by examining the degeneration of hippocampal neurons and the synthesis, release, transport, and metabolism of neurotransmitters. Our research aimed to provide a reference for improving the clinical application safety of Semen Strychni.

## Methods and Materials

### Chemicals and Reagents

The dried seeds of *Strychnos nux-vomica L* were purchased from SanXiang Chinese Herbal Medicine Co. Ltd., which was identified by associate professor Jinping Li, School of Pharmacy, Central South University. ISL was purchased from Chengdu Biopurify Phytochemicals, Ltd. (CAS NO.961-29-5, Chengdu, China). Rat monoamine oxidase (MAO) ELISA kit was obtained from CUSABIO (Catalog No.CSB-E15076r, Wuhan, China). Rat catechol-O-methyl transferase (COMT) ELISA kit was obtained from Jiangsu Meimian Industrial Co., Ltd. (Catalog No. MM-0833R1, Jiangsu, China). The standards of 13 neurotransmitters, including dopamine (DA), 3,4-dihydroxyphenylacetic acid (DOPAC), 5-hydroxytryptamine (5-HT), 5-hydroxyindole acetic acid (5-HIAA), homovanillic acid (HVA), norepinephrine (NE), epinephrine (E), tryptophan (Trp), tyrosine (Tyr), glutamate (Glu), glycine (Gly), γ-aminobutyric acid (GABA), and acetylcholine (ACh) were obtained from Sigma-Aldrich Chemicals (St. Louis, MO, United States). HPLC-grade acetonitrile and methanol were purchased from Merck (Darmstadt, Germany). Ultrapure water is made by a MilliQ water purification system (Millipore Corporation, Billerica, MA, United States).

### Preparation of Herbs

Extraction of Semen Strychni: after the raw Semen Strychni seeds were crushed, a proper amount of powder was taken and soaked in 75% acidic ethanol (pH = 5, 1:12, W/V) for 12 h, and heat to reflux for three times, each for 1 h. The extraction was filtered using a 0.45 μm microporous filter membrane under hot conditions. Then the filtrate was combined and concentrated by a rotary evaporator until all ethanol was volatilized. The filtrate pH was adjusted to 6.5 with 0.5 mol/L NaOH. The extract was mixed with 1% carboxymethylcellulose sodium (CMC-Na) solution and water to prepare 50 mg raw Semen Strychni/0.5 %CMC-Na ml. An appropriate amount of ISL was weighed and adjusted to 10 mg/ml in 0.5% CMC-Na solution. The composition of the Semen Strychni extract was detected by high-performance liquid chromatography coupled with tandem mass spectrometry and ultraviolet detector (HPLC-UV-MS) (Supplementary Material).

### Animal and Ethic

Male Sprague Dawley rats (200 ± 20 g) [Production license No.: SCXK (Xiang) 2019-0004] in SPF grade were purchased from Hunan SJA Laboratory Animal Co. Ltd. (China). Animals are raised in the barrier facilities of the Department of Laboratory Animals, Central South University [Experimental Animal Use Permit No.: SYXK (Xiang) 2015-0017]. The animals were acclimatized to a temperature-controlled environment (24–26°C), relative humidity (40–60%) under a 12/12 h light/dark cycle for a week with free access to food and water. This study was reviewed and approved by the Animal Care and Use Committee of Central South University [Ethics Approval No.:2021sydw0080]. All operations were conducted following the guidelines of the Declaration of Helsinki and the Guide for Care and Use of Laboratory Animals (Chinese Council).

### Experimental Design and Sample Collection

**FIGURE 1:**
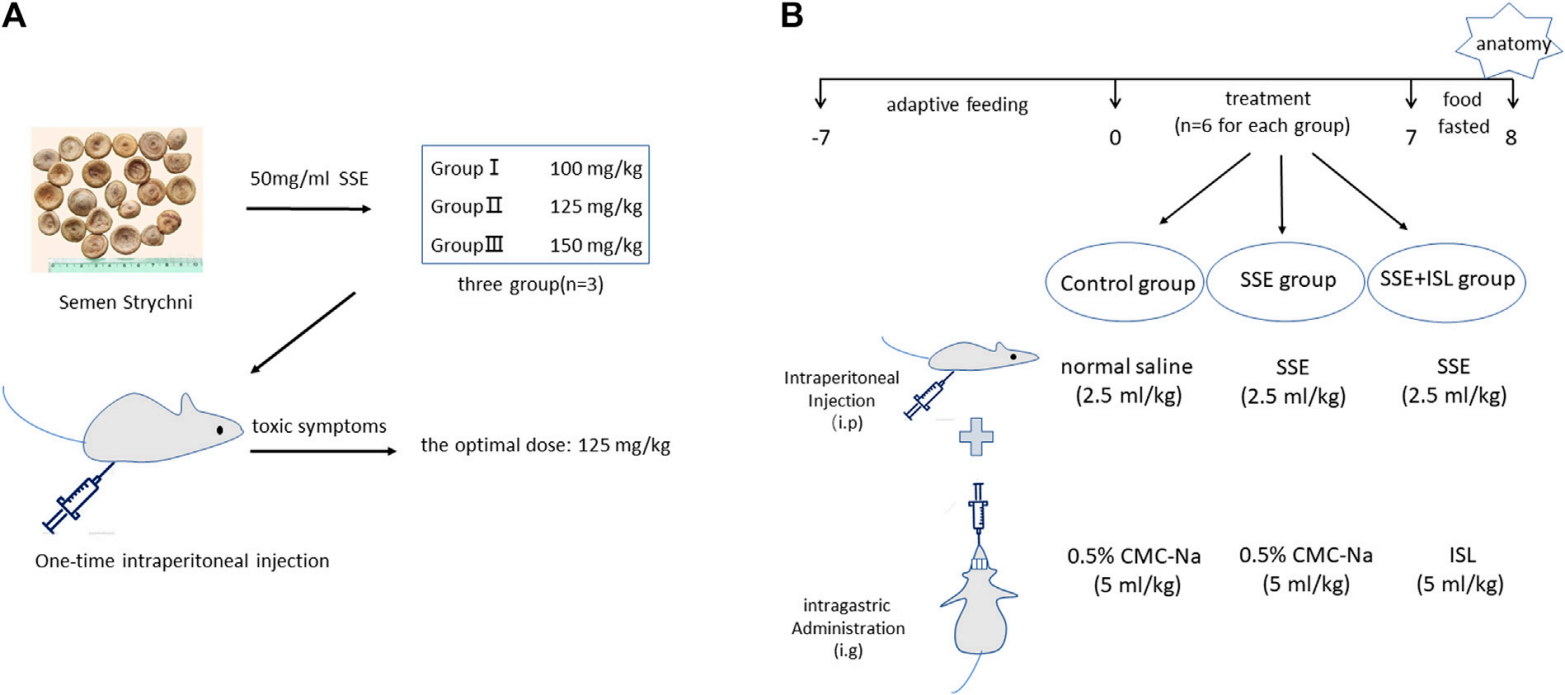
The maximally tolerated dose of rats was determined in pre-experiment **(A)**. Pre-experiment was employed to determine a dose that can cause poisoning but is not lethal. The animals were divided into three groups: SSE 100, 125, and 150 mg/kg, with three rats in each group. The experimental workflow for SSE intoxication and ISL detoxification **(B)**.

An overview of the experimental workflow is illustrated in Figure 1. First of all, pre-experiment was employed to determine a dose that can cause poisoning but is not lethal (Figure 1A). The animals were divided into three groups: SSE 100, 125, and 150 mg/kg, with three rats in each group. After intraperitoneal injection, the symptoms of nine rats were observed. Finally, a dose of 125 mg/kg was used. The dose volume was 2.5 ml/kg.

Second, 18 rats were randomly assigned to three groups (*n* = 6 for each) as follows (Figure 1B).

Control group (Control): 0.5% CMC-Na (5 ml/kg) was gavaged immediately after intraperitoneal injection of normal saline (2.5 ml/kg).

SSE group: 0.5% CMC-Na (5 ml/kg) was gavaged immediately after intraperitoneal injection of SSE (2.5 ml/kg, 125 mg/kg).

SSE + ISL group: ISL (5 ml/kg, 50 mg/kg) was gavaged immediately after intraperitoneal injection of SSE (2.5 ml/kg, 125 mg/kg).

According to the pre-experiment, the drug was administered continuously for 7 days according to the above administration methods, starting at 9 am. After the end of the 7th day, the food fasted for 12 h before anatomy.

At the time of dissection, the blood was gathered from the heart and transferred into the anticoagulation tube and coagulation tube, centrifuged (4°C, 3500 rpm) for 10 min. The supernatant was extracted to obtain plasma and serum. The wholly separated brain tissues of rats were randomly selected, soaked, and cleaned with normal saline several times and then put into a centrifuge tube with paraformaldehyde. The left and right hippocampus, hypothalamus, striatum, prefrontal cortex, and cerebellum were isolated. Serum, plasma, and brain tissue were stored at −80°C.

### Fluoro-Jade B Staining

FJ-B staining is a susceptive marker in the localization of neuronal degeneration (Schmued and Hopkins, 2000). In short, the process was as follows: The slices were successively put into xyleneⅠfor 15 min, xylene for 15 min, anhydrous ethanol for 5 min, anhydrous ethanol for 5 min, 85% ethanol for 5 min, 75% ethanol for 5 min, and then washed with distilled water. Add diluted FJ-B green fluorescent probe to the circled tissue with 50% glacial acetic acid as solvent and 1:400 FJ-B working solution at 4°C overnight. 4′,6-diamidino-2-phenylindole (DAPI) was dyed for 8 min, washed with pure water, dried with a hairdryer, xylene was transparent for 1 min, and the pieces were sealed with neutral resin. The sections were observed under an inverted Nikon fluorescence microscope (Nikon Eclipse C1, Japan), and the images were collected. (UV excitation wavelength 330–380 nm, emission wavelength 420 nm; The excitation wavelength of FITC green light is 465–495 nm, and the emission wavelength is 515–555 nm; Cy3 red light excitation wavelength 510–560 nm, emission wavelength 590 nm). The FJB-positive areas were quantified by thresholding the fluorescence intensity in these fluorescent images using ImageJ software.

### LC-MS/MS Method

LC-MS/MS method was developed to determine neurotransmitters in different brain regions, serum, and plasma. Experimental conditions refer to the literature (Shen et al., 2021). The mobile phase consisted of solvent A (0.2% formic acid and 5.0 mM ammonium formate in water) and solvent B (acetonitrile). The flow rate was set at 0.4 ml min^−1^, and the injection volume was 5 μl. LC-MS/MS analysis was performed in the following gradient elution mode: 0% B maintained at 0.01 min, increased to 20% at 0.1 min, increased to 25% at 2.3 min, increased to 50% at 5.31 min, and held for 1.0 min, increased to 95% at 7.0 min and held for 0.5 min, then decreased to 0% at 8.0 min followed by 5.0 min to achieve equilibrium. The operating parameters of the mass spectrometer were as follows: ion spray voltage, 5.0 kV; the source temperature of 550°C; curtain gas, 20 psi; CAD gas, medium; Ion source gas1, 55 psi; Ion source gas2, 50 psi. The residence time of each ion transition was set to 30 ms, and the electrospray ionization source was operated in positive mode.

### Quantitative Real-Time PCR

The total RNA from the hippocampus was extracted with TRIzol Reagent (Life, Carlsbad, CA, United States), and 1 μg total RNA was reverse-transcribed using Revert Aid First Strand cDNA Synthesis Kit (catalog no. #K1622; Thermo, Waltham, MA, United States). Real-time PCR was conducted using FastStart Universal SYBR Green Master (Rox) (Roche, Basel, Switzerland). The threshold cycle (CT) was determined using Bio-Rad CFX Manager 3.1 software. Relative quantification between compounds and untreated controls normalized to the levels of β-actin mRNA was calculated using the comparative CT method. Primers used for PCR, RT-PCR, and qRT-PCR are shown in Supplementary Table 1.

### Determination of MAO、COMT by ELISA

Blood from each sample was collected *in vivo*, centrifuged, and the supernatant was collected. COMT and MAO were determined using an enzyme-linked immunosorbent assay (ELISA) kit according to the manufacturer’s instructions. The optical density from each well was detected at 450 nm.

### Statistical Analysis

Data were represented as mean ± standard deviation (mean ± SD). and analyzed by SPSS 26.0 software. One-way ANOVA followed by a post least significant difference (LSD)-t test was used for statistical analysis. A value of *p* < 0.05 was considered statistical significance.

## Results

### The Maximum Tolerated Dose of Semen Strychni

To find the maximum tolerated dose of SSE, we did a preliminary experiment. The experimental results showed that rats in the 150 mg/kg group died of severe twitches, myotonia, and breathing difficulty after 2 min of administration. Rats in the 125 mg/kg group also showed similar symptoms, but the signs were relatively mild and could be spontaneously relieved. The 100 mg/kg group had only a mild convulsive reaction with a short duration. Thus, we found that 125 mg/kg/day was the maximum tolerated dose that can cause significant toxic effects for rats in the experiment.

### ISL Alleviated Semen Strychni-Induced Neurodegeneration in the Hippocampus

**FIGURE 2:**
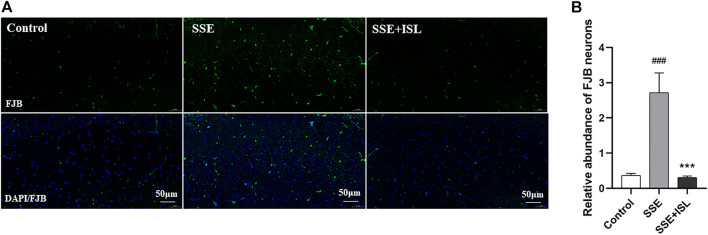
ISL alleviated Semen Strychni-induced neurodegeneration in the hippocampus. The immunofluorescence images **(A)** and respective relative IOD histograms **(B)** of FJB-positive neuronal cells in the CA1 region of rat brains. The density values are expressed in arbitrary units as the means ± standard deviation (mean ± SD). Significance: ###*p* < 0.001 (SSE group vs. Control group); ***p < 0.001 (SSE + ISL group vs. SSE group).

The pathological results were detected by FJ-B histofluorescence staining because it is a sensitive technique to detect neuronal degeneration. As shown in Figure 2, in the SSE group, Semen Strychni induced widespread neurodegeneration in the CA1 region of the hippocampus. Compared with the SSE group, in ISL group, there were few FJ-B positive neurons in CA1 region (Figure 2A). The relative integral optical density (IOD) histogram also showed significant growth in the SSE group. In contrast, the SSE + ISL group reduced the IOD values to almost the same as the Control group (Figure 2B). ###*p* < 0.001 (SSE group vs. Control group); ***p < 0.001 (SSE + ISL group vs. SSE group).

### Analyses of Neurotransmitters by LC-MS/MS

**FIGURE 3:**
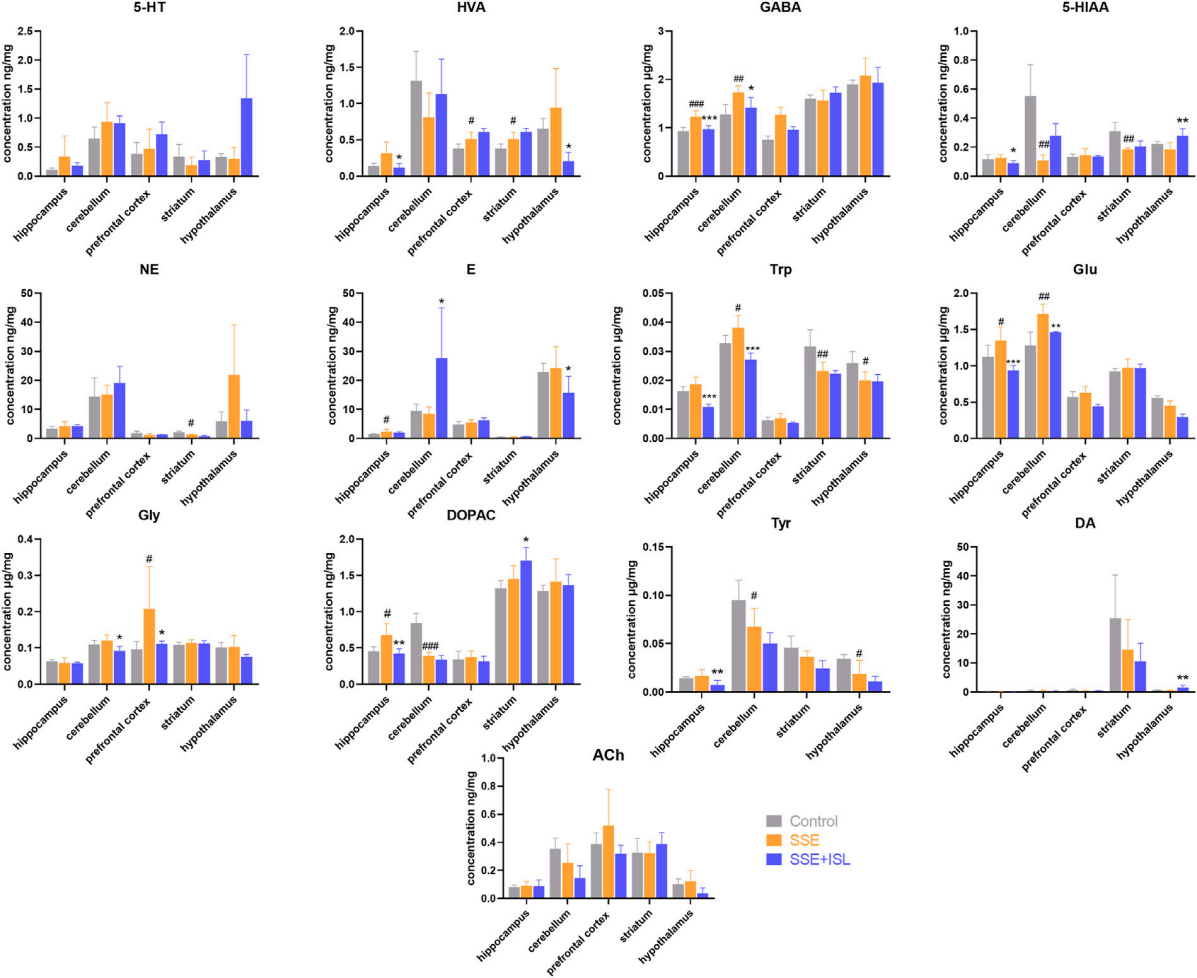
Determinations results of rat brain samples in different regions collected from different groups. Each value was represented as mean ± SD (standard deviation). ###*p* < 0.001, ##*p* < 0.01, and #*p* < 0.05 (SSE group vs. Control group); ***p < 0.001, **p < 0.01, and *p < 0.05 (SSE + ISL group vs. SSE group).

**FIGURE 4:**
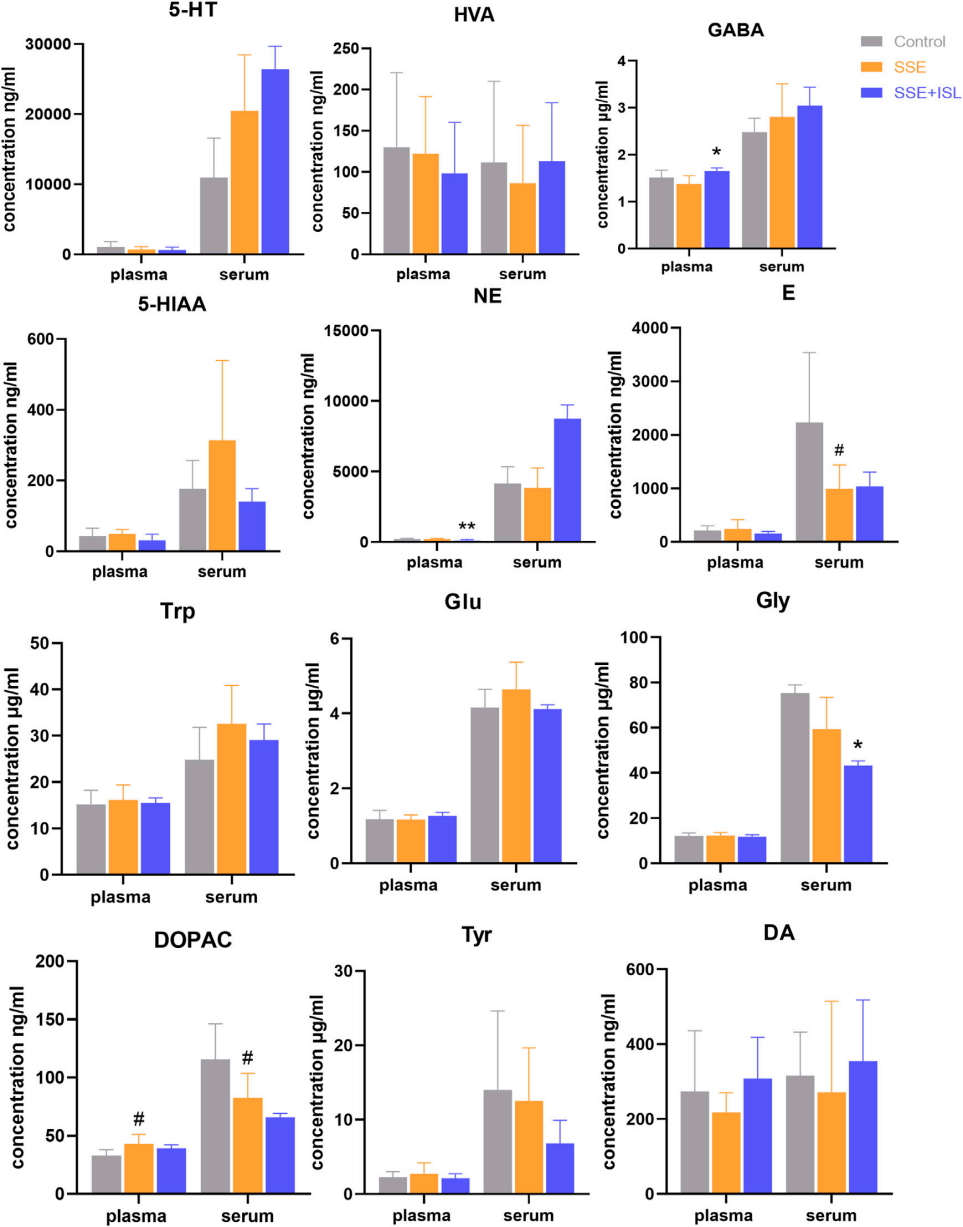
Determinations results of rat blood samples in plasma and serum collected from different groups. Each value was represented as mean ± SD (standard deviation). ###*p* < 0.001, ##*p* < 0.01, and #*p* < 0.05 (SSE group vs. Control group); ***p < 0.001, **p < 0.01, and *p < 0.05 (SSE + ISL group vs. SSE group).

Among the 13 neurotransmitters, compared with the Control group, the ones that showed significant changes in the SSE group were Glu and GABA in the hippocampus, cerebellum; DOPAC in the hippocampus, cerebellum, plasma, serum; 5-HIAA in the cerebellum, striatum; HVA in prefrontal cortex and striatum; E in the hippocampus, serum; Trp in the cerebellum, striatum, and hypothalamus; Tyr in the cerebellum and hypothalamus; NE in the striatum; Gly in the prefrontal cortex. And then some of them were returned to almost normal levels by ISL treatment, including Trp in the cerebellum, Glu in the hippocampus, cerebellum, Gly in the prefrontal cortex, GABA in the hippocampus, cerebellum, DOPAC in the hippocampus, NE in the hypothalamus, DA in serum. In summary, the number of neurotransmitters with significant differences in different regions were as follows: cerebellum > hippocampus = striatum = hypothalamus > serum > prefrontal cortex > plasma. The levels of neurotransmitters in the brain and blood are shown in Figures 3, 4. The concentration of these neurotransmitters showed a different trend in different regions. For example, the content of DOPAC increased significantly in the hippocampus and plasma, whereas a contrary tendency was observed in the cerebellum and serum. The expression of Trp was enhanced in the cerebellum but reduced in the striatum and hypothalamus. In addition, a marked increase in the concentration of E in hippocampus and a decrease in serum were discovered.

### Multiple Comparisons of Neurotransmitter Metabolic Pathways

**FIGURE 5:**
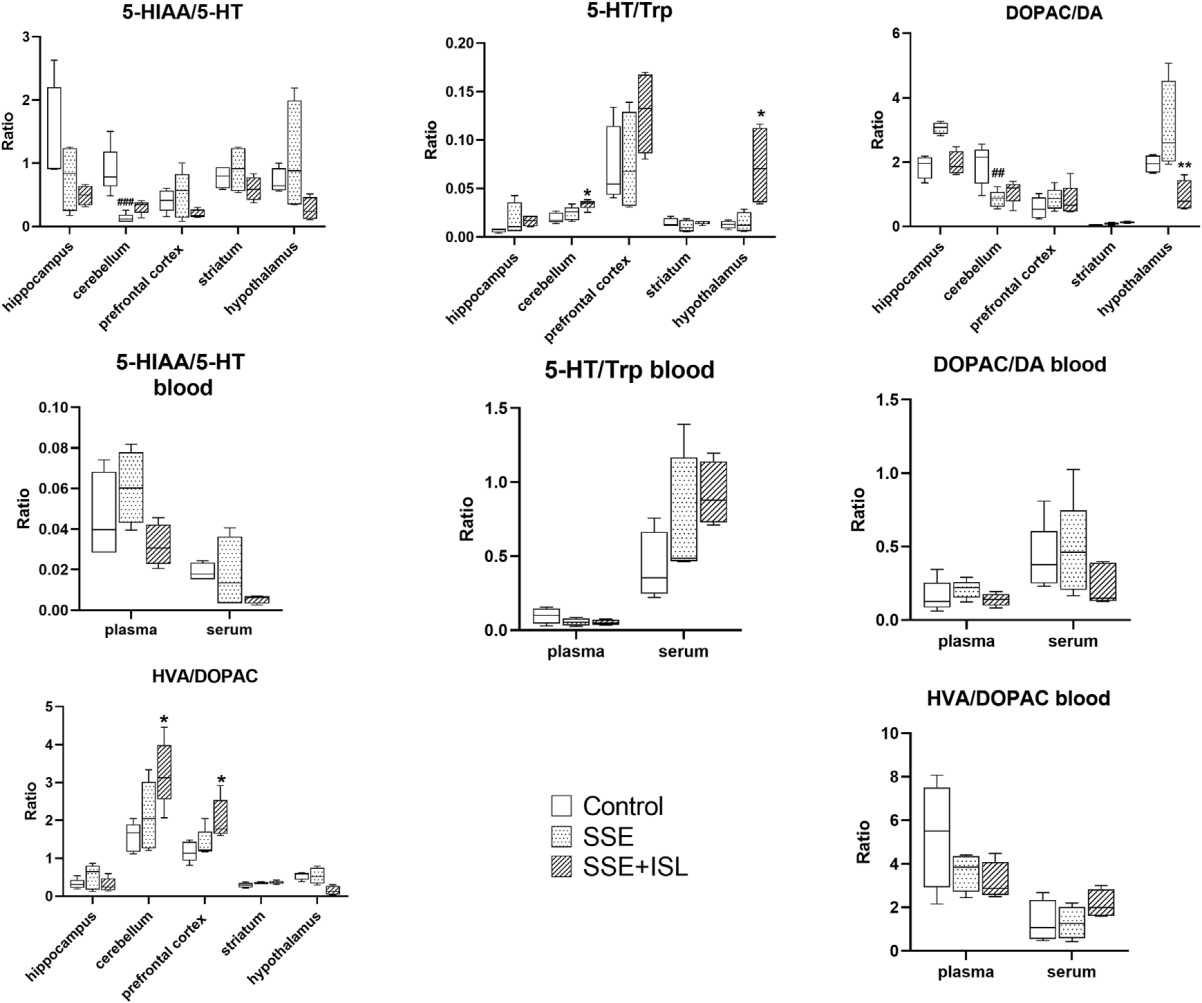
The ratios of product/neuroactive precursor in blood and brain samples. Each value was represented as mean ± SD (standard deviation). ###*p* < 0.001, ##*p* < 0.01, and #*p* < 0.05 (SSE group vs. Control group); ***p < 0.001, **p < 0.01, and *p < 0.05 (SSE + ISL group vs. SSE group)

The ratios of product/neuroactive precursor in blood and brain samples are shown in Figure 5. The 5-HIAA/5-HT and DOPAC/DA ratios in the cerebellum decreased significantly relative to the control group in the SSE group, while 5-HT/Trp and HVA/DOPAC in the brain and blood with no significant difference, suggesting that SSE restrained the metabolite conversions 5-HT and DA pathway to some extent.

### Changes in Neurotransmitter Receptors and Metabolic Enzymes

**FIGURE 6:**
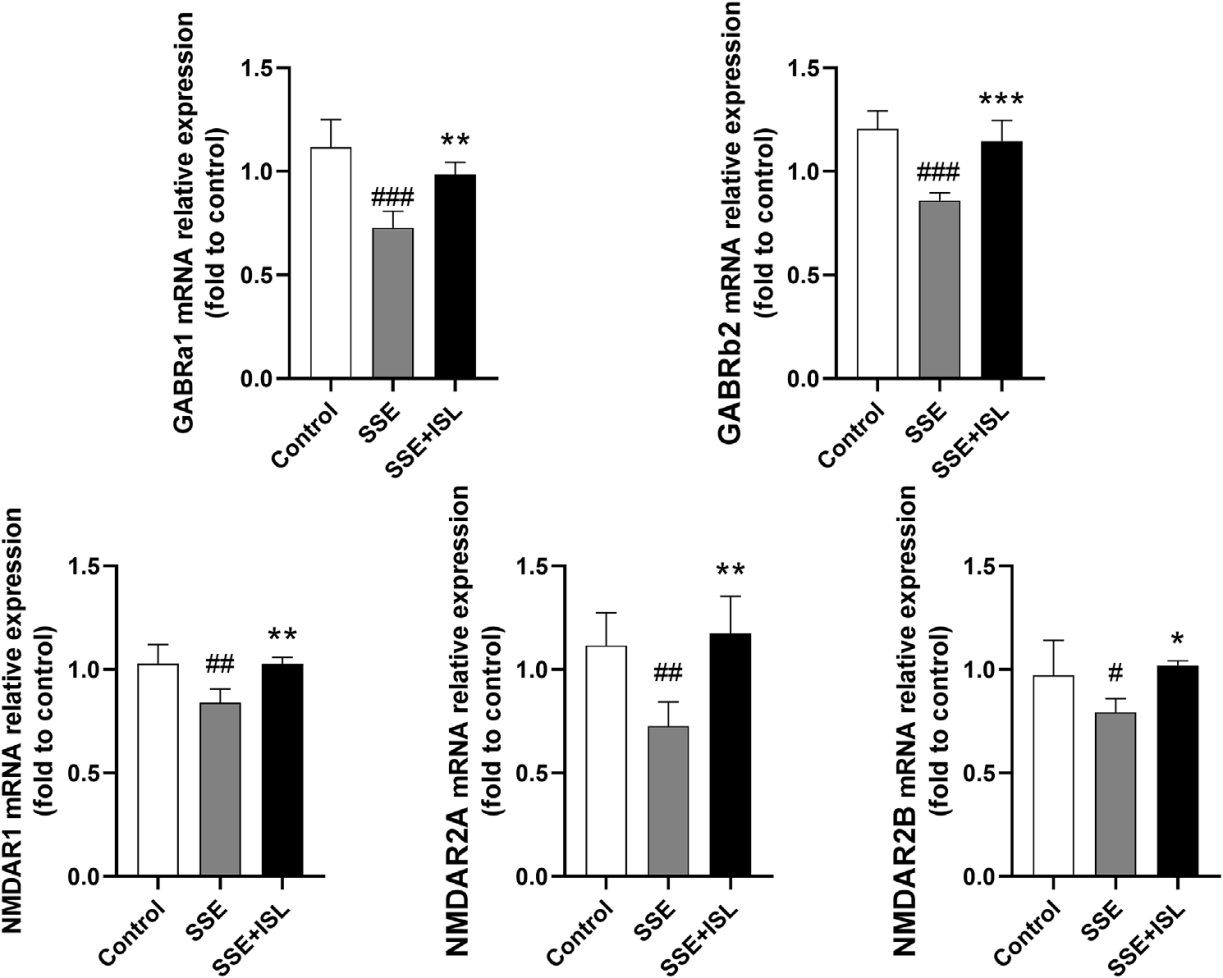
Quantitative analysis by qRT-PCR demonstrates the mRNA levels of NMDAR1, NMDAR2A, NMDAR2B, GABRa1, GABRb2 in the hippocampal homogenates. Values are presented as mean ± SD (*n* = 4). #*p* < 0.05 (SSE group vs. control group), *p < 0.05 (SSE + ISL group vs. SSE group).

**FIGURE 7:**
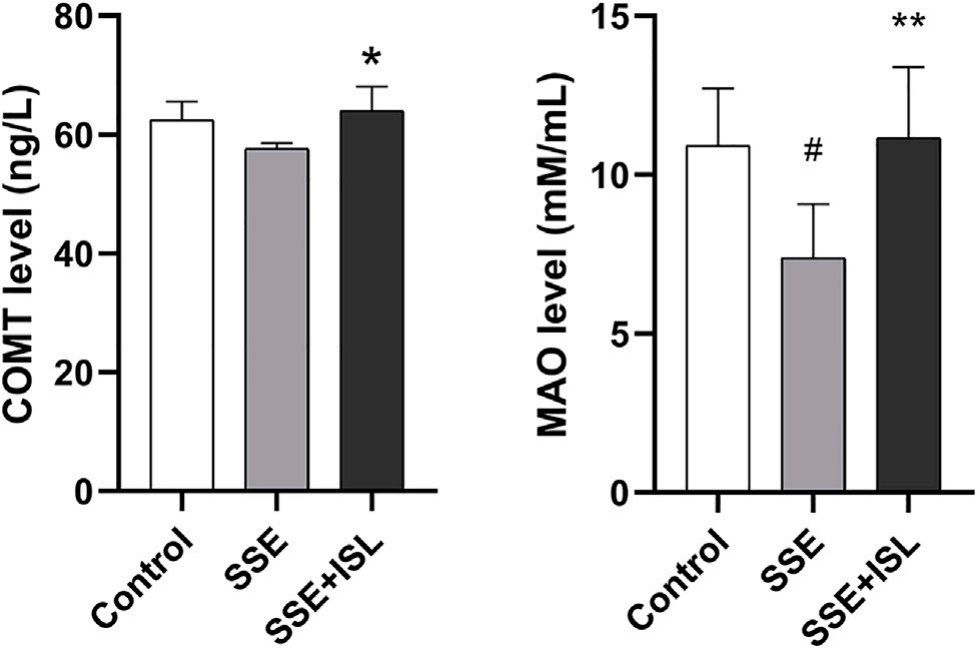
The neurotransmitter metabolic enzyme COMT and MAO were detected by enzyme-linked immunosorbent assay, and one duplicate hole was made. Data are expressed as the mean ± SD (*n* = 4–5). ##*p* < 0.01, and #*p* < 0.05 (SSE group vs. Control group); **p < 0.01, and *p < 0.05 (SSE + ISL group vs. SSE group).

To investigate the effect of SSE on the neurotransmitter metabolic pathway, we examined the receptors and metabolic enzymes. As shown in Figure 6, in the SSE group, the mRNA relative expression of GABRa1, GABRb2, NMDAR1, NMDAR2A, NMDAR2B exhibited a prominent downtrend compared with the control group. Intriguingly, ISL treatment increased the expression of SSE-induced receptors back to control levels. Besides, what we can see in Figure 7 was that the level of MAO and COMT significantly decreased in the SSE group and the SSE + ISL group in reverse. (###*p* < 0.001, ##*p* < 0.01, and #*p* < 0.05 (SSE group vs. Control group); ***p < 0.001, **p < 0.01, and *p < 0.05 (SSE + ISL group vs. SSE group).

## Discussion

In this study, the rats were observed for symptoms of SSE-induced neurotoxicity. By intraperitoneal injection of SSE (125 mg/kg) for seven consecutive days, severely neurons degenerated in the CA1 region. Neuronal injury impairs cognitive and motor function. Over time, damaged neurons impair hippocampal function, gradually leading to memory deficits (Lazarov and Hollands, 2016). Strychnine exposure altered neuronal synapses during embryonic development and impaired motor ability in adulthood (Roy et al., 2012). In our current study, neuronal degeneration was detected by FJ-B staining, which proved that Semen Strychni-induced neurotoxicity could affect the development of neurons and potentially cause cognitive impairment. Furthermore, ISL has a detoxification effect on SSE.

When severe Semen Strychni poisoning occurs, rats die of tonic seizures following clonic convulsions. With oral administration, it is rapidly absorbed in the gastrointestinal tract, and the first pass effect will reduce the amount of Semen Strychni entering the brain. At the same time, intraperitoneal injection induces clonic convulsions without subsequent tonic extension seizures and high Mortality Rates (Malone et al., 1992). Although studies have shown that rats orally administrated with SSE (552 mg/kg) for 15 d were found neuronal necrosis, intracellular lipid accumulation, and cell apoptosis in the cerebral cortex (Li et al., 2018; Sun et al., 2018). Our results indicated that compared with the oral administration for 15 days, the intraperitoneal administration for 7 days can evoke the symptoms of Semen Strychni neurotoxicity more quickly.

In addition to neuron damage, we also found changes in the concentration of neurotransmitters in different brain regions. According to our results, the number of neurotransmitters with significant differences in serum is higher than plasma. Therefore, detection of serum is sufficient. In addition, a comparison of significant changes in the number of neurotransmitters in the blood and the brain showed that changes in the serum were not representative of the brain because of the BBB (Gupta et al., 2019). The neurotoxicity may be more accurately studied by directly examining the relevant brain tissues (Ge et al., 2013).

In terms of amino acid neurotransmitters, in the SSE group, the concentrations of GABA and Glu in the cerebellum and hippocampus were significantly increased, which is consistent with the reports of Shi et al. (2017) and Sun et al. (2018). Glu is an excitatory neurotransmitter, and GABA is an inhibitory neurotransmitter. The comparative balance keeps the brain in a controlled environment. Once the balance is destroyed, the abnormal discharge of neurons will appear. The pathogenesis of convulsions is the relative imbalance of excitatory and inhibitory neurotransmitters (Akyuz et al., 2021). The symptoms of Semen Strychni poisoning showed that the excitatory effect of Glu was more significant than the inhibitory effect of GABA. In that case, the content of Glu in the synaptic cleft increases, its circulation is unbalanced, and a large amount of Ca^2+^ flows into neurons, which can cause Glu-induced neurotoxicity (Yang et al., 2012; Hu et al., 2018; Yang et al., 2020). Thus, we also examined the Glu receptors. Glutamatergic N-methyl-D-aspartic acid receptors (NMDARs, which are special ionic Glu receptors with Ca^2+^-gated channels) are essential receptors of Glu. It composes of NR1 (which binds the co-agonist glycine), NR2 (which binds glutamate), and NR3 subunits (Reis et al., 2009). NMDARs can mediate distinct cellular responses because of the regionalized receptor activities. Activation of synaptic NMDARs initiates plasticity and stimulates cell survival while activating extra-synaptic NMDARs promotes cell death (Wang and Reddy, 2017). In this study, the neurotoxicity of SSE inhibited the mRNA level of NR1, NR2A, NR2B, which probably resulted in degeneration of neurons and inhibition of glycine excitation.

GABA exists in the cerebellum, hippocampus (Yoon and Lee, 2014). There are two types of GABA receptors: GABA_A_ and GABA_B_ receptors (Brohan and Goudra, 2017). GABA mediates its effects through the ionotropic GABA_A_ receptors (GABRs), which belong to the Cys-loop superfamily of ligand-gated ion channels. The GABRs are pentameric, membrane-bound proteins surrounding an anion-selective pore (Giraudo et al., 2019). When GABA binds to receptors, it suppresses neuronal activity in the adult brain by opening a transmembrane channel permeable to chloride (Sigel and Steinmann, 2012; Zhu et al., 2018; Masiulis et al., 2019). However, in our results, the mRNA level of GABA receptors was decreased and could not bind with the increased GABA, thus the inhibitory effect of GABA on the neurotoxicity of Strychnos was weakened.

Besides, the concentrations of Tyr in the cerebellum and hypothalamus; Trp in the striatum and hypothalamus decreased, while Trp cerebellum increased in our results. Overall, the concentration of Trp went up (Sun et al., 2018), which indicates that the crucial role of Trp is in the cerebellum, and it tends to aggregate in the cerebellum (Sarna et al., 1991). Studies have described that when rats are given SSE, their exercise ability decreases (Shi et al., 2017). The surface area of the cerebellar cortex is 80% that of the cerebral cortex. The cerebellum has traditionally been thought to coordinate movement and maintain a sense of balance, but now research has shown that it also has a cognitive function (Koziol et al., 2014; Sereno et al., 2020; Van Overwalle et al., 2020). Once it’s damaged, there’s a lot of neurotransmitters that are affected. According to our results, the number of changes in neurotransmitters was highest in the cerebellum, followed by the hippocampus. The hippocampus is an important structure for learning and memory and is the site of a great deal of neurogenesis, it is also susceptible to biochemical and neurochemical alterations (Kang et al., 2017; Fernandes et al., 2020). In general, the structure and function of cerebellum and hippocampus were severely damaged when SSE was poisoned.

Trp and Tyr are involved in the two most important metabolic pathways of neurotransmitters--DA and Trp pathways. DA mainly exists in the striatum, providing learning signals by regulating synaptic plasticity (Berke, 2018; Walters et al., 2020). The striatum is associated with motor function—damage to the striatum results in aberrant sequencing of spontaneous movements and sensory-guided movements (Markowitz and Datta, 2020). Trp metabolism could occur both peripherally and centrally, and the 5-HT pathway is one of the main pathways (Li et al., 2020). Our study showed no significant changes in 5-HT and DA, but the concentration of NE, E, DOPAC, HVA, and 5-HIAA was significantly changed by neurotoxicity of SSE. The striatum and cerebellar endothelium may have the ability to convert Trp to 5-HIAA (Sarna et al., 1991), which is why the concentration of 5-HT was not affected. In addition, the ratio of 5-HIAA/5-HT and DOPAC/DA in the cerebellum and the activity of MAO were decreased, indicating that the inhibition of MAO inhibited 5-HT and DA metabolism. MAO is one of the main metabolic enzymes for DA metabolism. The downregulation of MAO leads to the decrease of DA metabolism (Graves et al., 2020). MAO is the essential metabolic enzyme to DA metabolism and catalyzes 5-HT to 5-HIAA (Wu et al., 2015). Previous reports demonstrated that Ca^2+^ selectively enhanced MAO activity in mice, rats, monkeys, and human Brains. But an antagonist of Ca^2+^ permeable NMDARs could potentially reduce MAO activity (Robinson et al., 2016). Therefore, inhibition of NMDARs mRNA expression by SSE neurotoxicity may be one of the reasons for MAO reduction.

Curiously, in our research, there was no significant change in the level of ACh, and the concentration of Gly was elevated in the prefrontal cortex in the SSE group. Ach is a central stimulant of the autonomic nervous system and mediated by cholinergic and nicotinic receptors. Increasing evidence suggests that the dysfunction of nicotinic acetylcholine receptor (nAChR), which is widely expressed in hippocampal and cortical neurons, may be related to the pathogenesis of epilepsy (Akyuz et al., 2021). The neurotoxicity of SSE may be due to its influence on nAChR, which is involved in epilepsy and affects Ach expression. Gly is a major inhibitory neurotransmitter in the brain stem and spinal cord. In the hippocampus, glycine synaptic plasticity is mainly controlled by the GlyT1 transporter. GlyT1 overexpression in the epileptic brain leads to dysregulation of Gly signaling (Shen et al., 2015). Neurotransmitters are unstable and quickly degraded (Hyman, 2005). Perhaps we can study the effect of SSE on glycine by examining the receptor GlyT1.

In the present study, oral administration of ISL (50 mg/kg) immediately after intraperitoneal injection of SSE attenuated the symptoms of severe twitches, myotonia, and breathing difficulty in rats. Previous studies found that oral administration of ISL (>25 mg/kg) has sedative-hypnotic effects by positive allosteric modulation of GABA_A_-BZD receptors (Cho et al., 2011). That is likely to be an important mechanism in the alleviation of ISL on Semen Strychni neurotoxicity. Additionally, ISL improved neuronal degeneration, restored the abnormal levels of neurotransmitters such as Gly, Trp, DOPAC, Glu, returned the mRNA expression of Glu, GABA receptors and the level of metabolic enzymes MAO, COMT to normal, which suggests that ISL has a detoxification effect.

ISL has memory enhancing and anti-Alzheimer’s activities, it can improve age-related neurodegenerative disorders (Ramalingam et al., 2018). ISL improved the degenerative neurons probably by inhibiting the mitochondrial protein mitoNEET (Chen et al., 2019) and reduced Trp expression by activating the extracellular signal-regulated protein kinase pathway (Lv et al., 2020). Moreover, excessively released Glu in the system can lead to the overstimulation of postsynaptic Glu receptors that leads to excitotoxic neuronal injury *in vitro* (Im et al., 2016). ISL can prevent Glu-induced toxicity by mitigating mitochondrial dysfunction (Ramalingam et al., 2018). The most important point is that ISL can act on receptors. ISL is a novel NMDA receptor antagonist that is bound to NMDA receptors to inhibit the Glu-induced increase in Ca^2+^ influx (Kawakami et al., 2011) and inhibits cocaine-induced striatal dopamine release by regulating the GABA_B_ receptor (Jang et al., 2008). Besides, Pan et al. found ISL inhibited the activity of MAO-A and MAO-B in brain mitochondria of normal rats (Pan et al., 2000). However, our results indicate the opposite. When damage occurs in the body, blood vessels, cells and other tissues will change (Wang and Li, 2018). Due to this, under normal circumstances, ISL reducing MAO activities, the opposite was observed in the case of Semen Strychni poisoning. We speculated that when ISL interacts with SSE, ISL has another neuroprotective mechanism of action to antagonize SSE-induced neurotoxicity. Further studies are required to confirm our speculation.

In summary, ISL has the function of regulating neurotransmitters and preventing damage to the BBB and neurons (Wang et al., 2008; Zhang et al., 2018b; Zhu et al., 2019b), which may be why ISL can detoxify the neurotoxicity of SSE. Therefore, the potential function and mechanism of the compatibility of ISL and Semen Strychni to alleviate the neurotoxicity of Semen Strychni deserve further study.

## Conclusion

We investigated the mechanism of Semen Strychni neurotoxicity and the detoxification mechanism of ISL from the perspective of neurotransmitter metabolic pathways. ISL has detoxifying effects when taken orally immediately after intraperitoneal injection of SSE by reducing the degeneration of neurons, restoring the metabolic pathways of Trp and DA. What’s innovative in our studies is that Semen Strychni-induced neurotoxicity suppressed the mRNA relative expression of GABRs and NMDARs, which not only reduced the activity of MAO and affected the metabolism of DA and 5-HT, but also may be one of the reasons for the neuronal degeneration and the inhibition of Gly excitation. In addition, we suggested that detecting the receptor GlyT1 can further investigate the effect of SSE on Gly. Taken together, from our studies, we speculated that Semen Strychni-induced neurotoxicity might risk neurological diseases. Intimately monitoring and timely rescue measures should be taken in clinical practice.

## Data Availability

The original contributions presented in the study are included in the article/Supplementary Material, further inquiries can be directed to the corresponding author.

## Supplementary Material

Supplementary Table 1 List of primers used for PCR, RT-PCR, and qRT-PCR. F: forward primer; R: reverse primer. Abbreviation: GAPDH: glyceraldehyde-3-phosphate dehydrogenase; GABRa1: gamma-aminobutyric acid type A receptor alpha1 subunit; NMDAR2A: ionotropic glutamate receptor NMDA type subunit 2A; Grin2b: NMDAR2B: ionotropic glutamate receptor NMDA type subunit 2B; Grin1:NMDAR1: glutamate ionotropic receptor NMDA type subunit 1; GABRb2: gamma-aminobutyric acid type A receptor subunit beta 2.

## References

[B1] Akyuz E. Polat A. K. Eroglu E. Kullu I. Angelopoulou E. Paudel Y. N. (2021). Revisiting the Role of Neurotransmitters in Epilepsy: An Updated Review. Life Sci. 265, 118826. 10.1016/j.lfs.2020.118826 33259863

[B2] Berke J. D. (2018). What Does Dopamine Mean? Nat. Neurosci. 21 (6), 787–793. 10.1038/s41593-018-0152-y 29760524 PMC6358212

[B3] Brohan J. Goudra B. G. (2017). The Role of GABA Receptor Agonists in Anesthesia and Sedation. CNS Drugs 31 (10), 845–856. 10.1007/s40263-017-0463-7 29039138

[B4] Chen X. Y. Ren H. H. Wang D. Chen Y. Qu C. J. Pan Z. H. (2019). Isoliquiritigenin Induces Mitochondrial Dysfunction and Apoptosis by Inhibiting mitoNEET in a Reactive Oxygen Species-dependent Manner in A375 Human Melanoma Cells. Oxid Med. Cell Longev 2019, 1–12. 10.1155/2019/9817576 PMC6360568 30805086

[B5] Cho S. Kim S. Jin Z. Yang H. Han D. Baek N. I. (2011). Isoliquiritigenin, a Chalcone Compound, Is a Positive Allosteric Modulator of GABAA Receptors and Shows Hypnotic Effects. Biochem. Biophys. Res. Commun. 413 (4), 637–642. 10.1016/j.bbrc.2011.09.026 21945440

[B6] Comai S. Bertazzo A. Brughera M. Crotti S. (2020). Tryptophan in Health and Disease. Adv. Clin. Chem. 95, 165–218. 10.1016/bs.acc.2019.08.005 32122523

[B7] Dimić D. Milenković D. Dimitrić Marković J. Marković Z. (2017). Antiradical Activity of Catecholamines and Metabolites of Dopamine: Theoretical and Experimental Study. Phys. Chem. Chem. Phys. 19 (20), 12970–12980. 10.1039/c7cp01716b 28480927

[B8] Fernandes R. M. Corrêa M. G. Aragão W. A. B. Nascimento P. C. Cartágenes S. C. Rodrigues C. A. (2020). Preclinical Evidences of Aluminum-Induced Neurotoxicity in hippocampus and Pre-frontal Cortex of Rats Exposed to Low Doses. Ecotoxicol Environ. Saf. 206, 111139. 10.1016/j.ecoenv.2020.111139 32861963

[B9] Fernando K. Jayasekara K. Warushahennadi J. Kumarasinghe I. Weerakoon K. Kularatne S. A. (2015). Intentional Ingestion of Strychnos Nux-Vomica Seeds Causing Severe Muscle Spasms and Cardiac Arrest: a Postmortem Report. Wilderness Environ. Med. 26 (1), 101–102. 10.1016/j.wem.2014.08.006 25712301

[B10] Gao Y. Lv X. Yang H. Peng L. Ci X. (2020). Isoliquiritigenin Exerts Antioxidative and Anti-inflammatory Effects via Activating the KEAP-1/Nrf2 Pathway and Inhibiting the NF-Κb and NLRP3 Pathways in Carrageenan-Induced Pleurisy. Food Funct. 11 (3), 2522–2534. 10.1039/c9fo01984g 32141447

[B11] Ge J. Wang J. Xue D. Zhu Z. Chen Z. Li X. (2013). Why Does a High-Fat Diet Induce Preeclampsia-like Symptoms in Pregnant Rats. Neural Regen. Res. 8 (20), 1872–1880. 10.3969/j.issn.1673-5374.2013.20.006 25206496 PMC4145971

[B12] Giraudo A. Krall J. Bavo F. Nielsen B. Kongstad K. T. Rolando B. (2019). Five-Membered N-Heterocyclic Scaffolds as Novel Amino Bioisosteres at γ-Aminobutyric Acid (GABA) Type A Receptors and GABA Transporters. J. Med. Chem. 62 (12), 5797–5809. 10.1021/acs.jmedchem.9b00026 31117514

[B13] Graves S. M. Xie Z. Stout K. A. Zampese E. Burbulla L. F. Shih J. C. (2020). Dopamine Metabolism by a Monoamine Oxidase Mitochondrial Shuttle Activates the Electron Transport Chain. Nat. Neurosci. 23 (1), 15–20. 10.1038/s41593-019-0556-3 31844313 PMC7257994

[B14] Gu L. Wang X. Liu Z. Ju P. Zhang L. Zhang Y. (2014). A Study of Semen Strychni-Induced Renal Injury and Herb-Herb Interaction of Radix Glycyrrhizae Extract And/or Rhizoma Ligustici Extract on the Comparative Toxicokinetics of Strychnine and Brucine in Rats. Food Chem. Toxicol. 68, 226–233. 10.1016/j.fct.2014.03.028 24704041

[B15] Guo R. Wang T. Zhou G. Xu M. Yu X. Zhang X. (2018). Botany, Phytochemistry, Pharmacology and Toxicity of Strychnos Nux-Vomica L. A Review. Am. J. Chin. Med. 46 (1), 1–23. 10.1142/S0192415X18500015 29298518

[B16] Gupta M. Lee H. J. Barden C. J. Weaver D. F. (2019). The Blood-Brain Barrier (BBB) Score. J. Med. Chem. 62 (21), 9824–9836. 10.1021/acs.jmedchem.9b01220 31603678

[B17] Hu X. Yang J. Sun Y. Gao X. Zhang L. Li Y. (2018). Lanthanum Chloride Impairs Memory in Rats by Disturbing the Glutamate-Glutamine Cycle and Over-activating NMDA Receptors. Food Chem. Toxicol. 113, 1–13. 10.1016/j.fct.2018.01.023 29353069

[B18] Hyman S. E. (2005). Neurotransmitters. Curr. Biol. 15 (5), R154–R158. 10.1016/j.cub.2005.02.037 15753022

[B19] Im K. H. Nguyen T. K. Choi J. Lee T. S. (2016). *In Vitro* Antioxidant, Anti-diabetes, Anti-dementia, and Inflammation Inhibitory Effect of Trametes Pubescens Fruiting Body Extracts. Molecules 21 (5). 10.3390/molecules21050639 PMC6273937 27196881

[B20] Jang E. Y. Choe E. S. Hwang M. Kim S. C. Lee J. R. Kim S. G. (2008). Isoliquiritigenin Suppresses Cocaine-Induced Extracellular Dopamine Release in Rat Brain through GABA(B) Receptor. Eur. J. Pharmacol. 587 (1-3), 124–128. 10.1016/j.ejphar.2008.03.054 18495107

[B21] Kang E. Berg D. A. Furmanski O. Jackson W. M. Ryu Y. K. Gray C. D. (2017). Neurogenesis and Developmental Anesthetic Neurotoxicity. Neurotoxicol Teratol 60, 33–39. 10.1016/j.ntt.2016.10.001 27751818 PMC5541260

[B22] Kawakami Z. Ikarashi Y. Kase Y. (2011). Isoliquiritigenin Is a Novel NMDA Receptor Antagonist in Kampo Medicine Yokukansan. Cell Mol Neurobiol 31 (8), 1203–1212. 10.1007/s10571-011-9722-1 21691759 PMC11498536

[B23] Koziol L. F. Budding D. Andreasen N. D'Arrigo S. Bulgheroni S. Imamizu H. (2014). Consensus Paper: the Cerebellum's Role in Movement and Cognition. Cerebellum 13 (1), 151–177. 10.1007/s12311-013-0511-x 23996631 PMC4089997

[B24] Lazarov O. Hollands C. (2016). Hippocampal Neurogenesis: Learning to Remember. Prog. Neurobiol. 138-140, 1–18. 10.1016/j.pneurobio.2015.12.006 26855369 PMC4828289

[B25] Li C. C. Jiang N. Gan L. Zhao M. J. Chang Q. Liu X. M. (2020). Peripheral and Cerebral Abnormalities of the Tryptophan Metabolism in the Depression-like Rats Induced by Chronic Unpredicted Mild Stress. Neurochem. Int. 138, 104771. 10.1016/j.neuint.2020.104771 32450184

[B26] Li S. Chu Y. Zhang R. Sun L. Chen X. (2018). Prophylactic Neuroprotection of Total Glucosides of Paeoniae Radix Alba against Semen Strychni-Induced Neurotoxicity in Rats: Suppressing Oxidative Stress and Reducing the Absorption of Toxic Components. Nutrients 10 (4), 514. 10.3390/nu10040514 PMC5946299 29677121

[B27] Li Y. Wang J. Xiao Y. Wang Y. Chen S. Yang Y. (2015). A Systems Pharmacology Approach to Investigate the Mechanisms of Action of Semen Strychni and Tripterygium Wilfordii Hook F for Treatment of Rheumatoid Arthritis. J. Ethnopharmacol 175, 301–314. 10.1016/j.jep.2015.09.016 26386382

[B28] Lin A. Su X. She D. Qiu K. He Q. Liu Y. (2016). LC-MS/MS Determination and Comparative Pharmacokinetics of Strychnine, Brucine and Their Metabolites in Rat Plasma after Intragastric Administration of Each Monomer and the Total Alkaloids from Semen Strychni. J. Chromatogr. B Analyt Technol. Biomed. Life Sci. 1008, 65–73. 10.1016/j.jchromb.2015.11.012 26625339

[B29] Lu L. Huang R. Wu Y. Jin J. M. Chen H. Z. Zhang L. J. (2020). Brucine: A Review of Phytochemistry, Pharmacology, and Toxicology. Front. Pharmacol. 11, 377. 10.3389/fphar.2020.00377 32308621 PMC7145893

[B30] Lv J. Fu Y. Cao Y. Jiang S. Yang Y. Song G. (2020). Isoliquiritigenin Inhibits Melanogenesis, Melanocyte Dendricity and Melanosome Transport by Regulating ERK-Mediated MITF Degradation. Exp. Dermatol. 29 (2), 149–157. 10.1111/exd.14066 31785162

[B31] Malone M. H. St John-Allan K. M. Bejar E. (1992). Brucine Lethality in Mice. J. Ethnopharmacol 35 (3), 295–297. 10.1016/0378-8741(92)90028-p 1347799

[B32] Markowitz J. E. Datta S. R. (2020). The Striatum Specifies the Statistics of Behavior. Neuropsychopharmacology 45 (1), 222–223. 10.1038/s41386-019-0493-6 31467423 PMC6879596

[B33] Masiulis S. Desai R. Uchański T. Serna Martin I. Laverty D. Karia D. (2019). GABAA Receptor Signalling Mechanisms Revealed by Structural Pharmacology. Nature 565 (7740), 454–459. 10.1038/s41586-018-0832-5 30602790 PMC6370056

[B34] Matsubara K. Watabe H. Kumakura Y. Hayashi T. Endres C. J. Minato K. (2011). Sensitivity of Kinetic Macro Parameters to Changes in Dopamine Synthesis, Storage, and Metabolism: a Simulation Study for [¹⁸F]FDOPA PET by a Model with Detailed Dopamine Pathway. Synapse 65 (8), 751–762. 10.1002/syn.20899 21190220

[B35] Mutolo D. Cinelli E. Bongianni F. Pantaleo T. (2011). Identification of a Cholinergic Modulatory and Rhythmogenic Mechanism within the Lamprey Respiratory Network. J. Neurosci. 31 (37), 13323–13332. 10.1523/JNEUROSCI.2764-11.2011 21917815 PMC6623278

[B36] Naik B. S. Chakrapani M. (2009). A Rare Case of Brucine Poisoning Complicated by Rhabdomyolysis and Acute Renal Failure. Malays J. Pathol. 31 (1), 67–69. 19694317

[B37] Pan X. Kong L. D. Zhang Y. Cheng C. H. Tan R. X. (2000). *In Vitro* inhibition of Rat Monoamine Oxidase by Liquiritigenin and Isoliquiritigenin Isolated from Sinofranchetia Chinensis. Acta Pharmacol. Sin 21 (10), 949–953. 11501051

[B38] Ramalingam M. Kim H. Lee Y. Lee Y. I. (2018). Phytochemical and Pharmacological Role of Liquiritigenin and Isoliquiritigenin from Radix Glycyrrhizae in Human Health and Disease Models. Front. Aging Neurosci. 10, 348. 10.3389/fnagi.2018.00348 30443212 PMC6221911

[B39] Reis H. J. Guatimosim C. Paquet M. Santos M. Ribeiro F. M. Kummer A. (2009). Neuro-transmitters in the central Nervous System & Their Implication in Learning and Memory Processes. Curr. Med. Chem. 16 (7), 796–840. 10.2174/092986709787549271 19275596

[B40] Ren T. Li M. Zheng H. Liu W. Zhang J. (2018). Microdialysis Combined with RRLC-MS/MS for the Pharmacokinetics of Two Major Alkaloids of Bi Qi Capsule and the Potential Roles of P-Gp and BCRP on Their Penetration. J. Chromatogr. B Analyt Technol. Biomed. Life Sci. 1092, 72–81. 10.1016/j.jchromb.2018.05.048 29883892

[B41] Robinson B. L. Dumas M. Cuevas E. Gu Q. Paule M. G. Ali S. F. (2016). Distinct Effects of Ketamine and Acetyl L-Carnitine on the Dopamine System in Zebrafish. Neurotoxicol Teratol 54, 52–60. 10.1016/j.ntt.2016.02.004 26898327 PMC4924529

[B42] Roy N. M. Arpie B. Lugo J. Linney E. Levin E. D. Cerutti D. (2012). Brief Embryonic Strychnine Exposure in Zebrafish Causes Long-Term Adult Behavioral Impairment with Indications of Embryonic Synaptic Changes. Neurotoxicol Teratol 34 (6), 587–591. 10.1016/j.ntt.2012.08.001 23022260 PMC4338986

[B43] Sarna G. S. Hutson P. H. O'Connell M. T. Curzon G. (1991). Effect of Tryptophan on Extracellular Concentrations of Tryptophan and 5-hydroxyindoleacetic Acid in the Striatum and Cerebellum. J. Neurochem. 56 (5), 1564–1568. 10.1111/j.1471-4159.1991.tb02052.x 1707438

[B44] Schmued L. C. Hopkins K. J. (2000). Fluoro-Jade B: a High Affinity Fluorescent Marker for the Localization of Neuronal Degeneration. Brain Res. 874 (2), 123–130. 10.1016/s0006-8993(00)02513-0 10960596

[B45] Sereno M. I. Diedrichsen J. Tachrount M. Testa-Silva G. d'Arceuil H. De Zeeuw C. (2020). The Human Cerebellum Has Almost 80% of the Surface Area of the Neocortex. Proc. Natl. Acad. Sci. U S A. 117 (32), 19538–19543. 10.1073/pnas.2002896117 32723827 PMC7431020

[B46] Shen H. Y. van Vliet E. A. Bright K. A. Hanthorn M. Lytle N. K. Gorter J. (2015). Glycine Transporter 1 Is a Target for the Treatment of Epilepsy. Neuropharmacology 99, 554–565. 10.1016/j.neuropharm.2015.08.031 26302655 PMC4655139

[B47] Shen J. Wang H. Huang H. Li H. Li C. Yan C. (2021). Absolute Quantitative Analysis of Endogenous Neurotransmitters and Amino Acids by Liquid Chromatography-Tandem Mass Spectrometry Combined with Multidimensional Adsorption and Collision Energy Defect. J. Chromatogr. A. 1638, 461867. 10.1016/j.chroma.2020.461867 33485029

[B48] Shi D. Yang J. Jiang Y. Wen L. Wang Z. Yang B. (2020). The Antioxidant Activity and Neuroprotective Mechanism of Isoliquiritigenin. Free Radic. Biol. Med. 152, 207–215. 10.1016/j.freeradbiomed.2020.03.016 32220625

[B49] Shi H. Hou C. Gu L. Xing H. Zhang M. Zhao L. (2017). Investigation of the Protective Effect of Paeonia Lactiflora on Semen Strychni-Induced Neurotoxicity Based on Monitoring Nine Potential Neurotoxicity Biomarkers in Rat Serum and Brain Tissue. Metab. Brain Dis. 32 (1), 133–145. 10.1007/s11011-016-9894-y 27521025

[B50] Sigel E. Steinmann M. E. (2012). Structure, Function, and Modulation of GABA(A) Receptors. J. Biol. Chem. 287 (48), 40224–40231. 10.1074/jbc.R112.386664 23038269 PMC3504738

[B51] Spiering M. J. (2018). The Discovery of GABA in the Brain. J. Biol. Chem. 293 (49), 19159–19160. 10.1074/jbc.CL118.006591 30530855 PMC6295731

[B52] Sun L. Chen Y. Hou C. Sun X. Wang Z. Li S. (2018). Neuroprotective Effect of Total Glycosides from Paeonies against Neurotoxicity Induced by Strychnos Alkaloids Related to Recovering the Levels of Neurotransmitters and Neuroendocrine Hormones in Rat Serum and Brain. RSC Adv. 8 (51), 29210–29219. 10.1039/c8ra05384g PMC9084482 35548016

[B53] Tian J. Hu J. Liu D. Yin J. Chen M. Zhou L. (2021). Cadmium Chloride-Induced Transgenerational Neurotoxicity in Zebrafish Development. Environ. Toxicol. Pharmacol. 81, 103545. 10.1016/j.etap.2020.103545 33171223

[B54] Van Overwalle F. Van de Steen F. van Dun K. Heleven E. (2020). Connectivity between the Cerebrum and Cerebellum during Social and Non-social Sequencing Using Dynamic Causal Modelling. Neuroimage 206, 116326. 10.1016/j.neuroimage.2019.116326 31678499

[B55] Vogt N. (2018). Detecting Acetylcholine. Nat. Methods 15 (9), 648. 10.1038/s41592-018-0131-y 30171237

[B56] Vogt N. (2019). Sensing Neurotransmitters. Nat. Methods 16 (1), 17. 10.1038/s41592-018-0268-8 30573837

[B57] Walters S. H. Shu Z. Michael A. C. Levitan E. S. (2020). Regional Variation in Striatal Dopamine Spillover and Release Plasticity. ACS Chem. Neurosci. 11 (6), 888–899. 10.1021/acschemneuro.9b00577 32073248 PMC9668542

[B58] Wang K. Li C. (2018). Effects of Dexmedetomidine on Inflammatory Factors, T Lymphocyte Subsets and Expression of NF-Κb in Peripheral Blood Mononuclear Cells in Patients Receiving Radical Surgery of colon Carcinoma. Oncol. Lett. 15 (5), 7153–7157. 10.3892/ol.2018.8205 29725437 PMC5920235

[B59] Wang R. Reddy P. H. (2017). Role of Glutamate and NMDA Receptors in Alzheimer's Disease. J. Alzheimers Dis. 57 (4), 1041–1048. 10.3233/JAD-160763 27662322 PMC5791143

[B60] Wang W. Hu X. Zhao Z. Liu P. Hu Y. Zhou J. (2008). Antidepressant-like Effects of Liquiritin and Isoliquiritin from Glycyrrhiza Uralensis in the Forced Swimming Test and Tail Suspension Test in Mice. Prog. Neuropsychopharmacol. Biol. Psychiatry 32 (5), 1179–1184. 10.1016/j.pnpbp.2007.12.021 18289754

[B61] Wang Y. Kim S. C. Wu T. Jiao Y. Jin H. Hyo Lee B. (2020). Isoliquiritigenin Attenuates Anxiety-like Behavior and Locomotor Sensitization in Rats after Repeated Exposure to Nicotine. Evid. Based Complement. Alternat Med. 2020, 9692321. 10.1155/2020/9692321 32256666 PMC7102418

[B62] Wu J. P. Li M. H. Chen J. S. Chung S. Y. Lee H. L. (2015). Disturbances to Neurotransmitter Levels and Their Metabolic Enzyme Activity in a Freshwater Planarian Exposed to Cadmium. Neurotoxicology 47, 72–81. 10.1016/j.neuro.2015.01.003 25644215

[B63] Wu M. Fang M. Hu Y. Wang X. (2012). Four Types of Traditional Chinese Medicine Inducing Epileptic Seizures. Seizure 21 (5), 311–315. 10.1016/j.seizure.2012.02.010 22475771

[B64] Yang E. J. Min J. S. Ku H. Y. Choi H. S. Park M. K. Kim M. K. (2012). Isoliquiritigenin Isolated from Glycyrrhiza Uralensis Protects Neuronal Cells against Glutamate-Induced Mitochondrial Dysfunction. Biochem. Biophys. Res. Commun. 421 (4), 658–664. 10.1016/j.bbrc.2012.04.053 22538371

[B65] Yang T. Xu Z. Liu W. Xu B. Deng Y. (2020). Oxidative Stress Accelerates Synaptic Glutamate Dyshomeostasis and NMDARs Disorder during Methylmercury-Induced Neuronal Apoptosis in Rat Cerebral Cortex. Environ. Toxicol. 35 (6), 683–696. 10.1002/tox.22904 32061141

[B66] Yao J. Wang J. Wu L. Lu H. Wang Z. Yu P. (2020). Perinatal Exposure to Bisphenol A Causes a Disturbance of Neurotransmitter Metabolic Pathways in Female Mouse Offspring: A Focus on the Tryptophan and Dopamine Pathways. Chemosphere 254, 126715. 10.1016/j.chemosphere.2020.126715 32334245

[B67] Yoon B. E. Lee C. J. (2014). GABA as a Rising Gliotransmitter. Front. Neural Circuits 8, 141. 10.3389/fncir.2014.00141 25565970 PMC4269106

[B68] Zhan C. Yang J. (2006). Protective Effects of Isoliquiritigenin in Transient Middle Cerebral Artery Occlusion-Induced Focal Cerebral Ischemia in Rats. Pharmacol. Res. 53 (3), 303–309. 10.1016/j.phrs.2005.12.008 16459097

[B69] Zhang M. Deng Y. Wang C. Cai H. L. Wen J. Fang P. F. (2018a). An LC-MS/MS Method for Determination of Bioactive Components of Liquorice and Semen Strychni in Rat Plasma: Application to a Pharmacokinetics Study. Drug Test. Anal. 10 (2), 262–271. 10.1002/dta.2210 28447397

[B70] Zhang M. Wu Y. Xie L. Teng C. H. Wu F. F. Xu K. B. (2018b). Isoliquiritigenin Protects against Blood-brain B-arrier D-amage and I-nhibits the S-ecretion of P-ro-inflammatory C-ytokines in M-ice after T-raumatic B-rain I-njury. Int. Immunopharmacol 65, 64–75. 10.1016/j.intimp.2018.09.046 30290368

[B71] Zhu S. Noviello C. M. Teng J. Walsh R. M. Jr. Kim J. J. Hibbs R. E. (2018). Structure of a Human Synaptic GABAA Receptor. Nature 559 (7712), 67–72. 10.1038/s41586-018-0255-3 29950725 PMC6220708

[B72] Zhu X. Liu J. Chen O. Xue J. Huang S. Zhu W. (2019a). Neuroprotective and Anti-inflammatory Effects of Isoliquiritigenin in Kainic Acid-Induced Epileptic Rats via the TLR4/MYD88 Signaling Pathway. Inflammopharmacology 27 (6), 1143–1153. 10.1007/s10787-019-00592-7 31037573

[B73] Zhu X. Liu J. Huang S. Zhu W. Wang Y. Chen O. (2019b). Neuroprotective Effects of Isoliquiritigenin against Cognitive Impairment via Suppression of Synaptic Dysfunction, Neuronal Injury, and Neuroinflammation in Rats with Kainic Acid-Induced Seizures. Int. Immunopharmacol 72, 358–366. 10.1016/j.intimp.2019.04.028 31030091

