# Supplementary material for: Isoliquiritigenin Alleviates Semen Strychni-Induced Neurotoxicity by Restoring the Metabolic Pathway of Neurotransmitters in Rats
Source: Front Pharmacol. 2021 Nov 15;12:762290. doi: 10.3389/fphar.2021.762290 (PMC8634445; doi:10.3389/fphar.2021.762290)
Supplement: Supplementary file 1 [file DataSheet1.docx]

Supplementary Material

# Supplementary Methods and materials

## Chemicals and reagents

HPLC ( LC-20ADVP, Shimadzu, Kyoto, Japan).AB SCIEX 4000 QTRAP( AB SCIEX , USA). Analyst software workstation (AB SCIEX, USA). The standards of brucine and [strychnine](javascript:;) were obtained from China National Institutes for Food and Drug Control (Lot number: 110706-200506;11075-200306)

## HPLC-UV-MS

**1.2.1 Chromatographic conditions**

The instrument was fitted with a Sino ChromeODS-BP( 4.6 mm × 250 mm, 5 μm); mobile phase A was water containing 0.1% formic acid and mobile phase B was acetonitrile ; flow rate, 0.3 mL min^-1^; column temperature, 40 °C; injection volume, 5 µL; and gradient elution program (0.01 min, 8% B; 2min, 8% B; 30 min, 25% B;32 min, 30% B; 50 min, 45% B; 53 min, 85% B; 60 min, 85% B; 61 min, 8% B; and ended at 77 min). UV detection was done at 254 nm.

**1.2.2 Mass spectrometry conditions**

Positive ion scanning was applied to the test solution of Semen Strychni extract, the scan mode was full scan and the scan range was m/z 50-1000. The ion source was an electrospray ionization source (ESI), the collision gas was helium, source temperature 120℃, desolvation temperature 400°C. The desolvation and cone gas flow rates were 750 L/h and 50 L/h. The cone voltage was 30 V for  ESI+, capillary voltage 2.5 V.

# Supplementary Figures and Tables

## Supplementary Figures


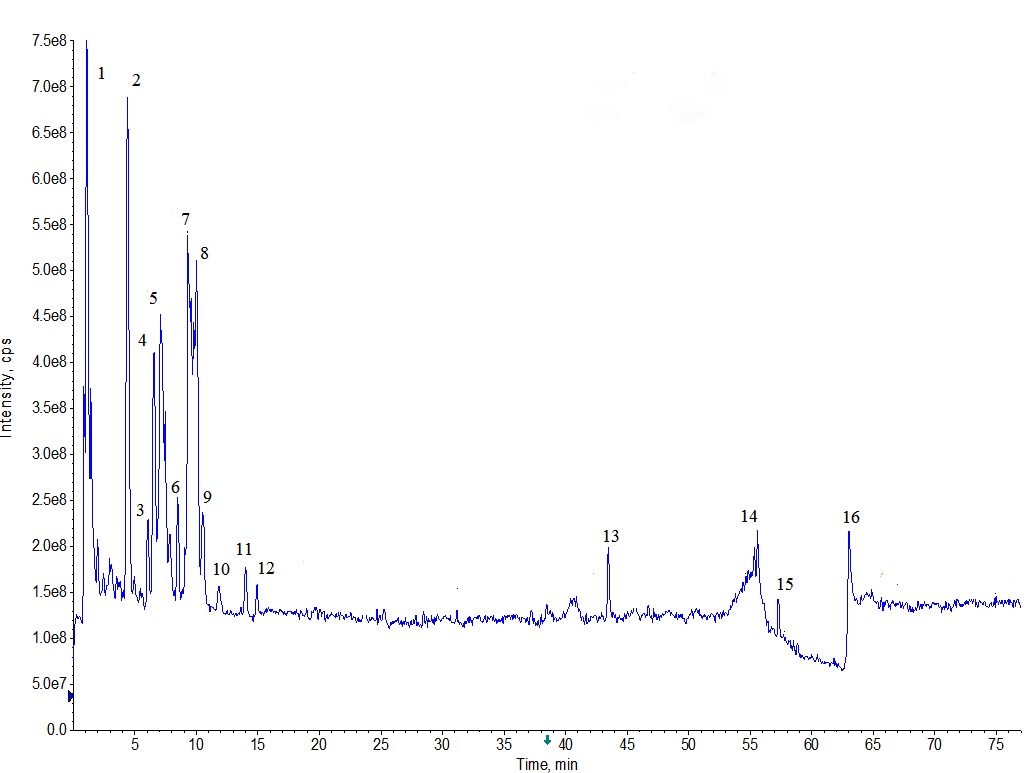


**Supplementary Figure 1.** The chromatograms of Semen Strychni extract.

## Supplementary Tables

**Supplementary Table 1** Mass spectrum analysis of components in Semen Strychni extract

| Peak | Retention time | Fragment ions(m/z) | | Identification | |
| --- | --- | --- | --- | --- | --- |
| 1 | 1.18 | 365,204,184 | - | | |
| 2 | 4.36 | 399,382,237,219,184 | - | |  |
| 3 | 6.07 | 349,264,184,155 | 9-N-methylstrychnine | |  |
| 4 | 6.56 | 377,360,215,184 | - | |  |
| 5 | 7.50 | 335,264,184,155 | Strychnine | |  |
| 6 | 8.52 | 409,325,263,198,119 | 2,3-Dimethoxy-19-N-methylstrychnine | |  |
| 7 | 9.66 | 395,369,326,264,198 | Brucine | |  |
| 8 | 9.94 | 365,295,214,185 | 3-Methoxystrychnine | |  |
| 9 | 10.17 | 365,294,214,185 | 4-Methoxystrychnine | |  |
| 10 | 10.57 | 365,338,295,214,186 | β-Colubrine | |  |
| 11 | 14.01 | 443,346,248,220,183 | - | |  |
| 12 | 14.94 | 503,475,325,280,229,151 | - | |  |
| 13 | 43.50 | 454,436,408,189,174 | - | |  |
| 14 | 55.62 | 148,120,63 | - | |  |
| 15 | 57.29 | 339,304,148,93,84 | - | |  |
| 16 | 63.07 | 804,746,622,489,314 | - | |  |

Note: - means unable to be speculated and confirmed
